# Propensity score matching analysis of the prognosis for the rare oxyphilic subtype of thyroid cancer (Hurthle cell carcinoma)

**DOI:** 10.18632/oncotarget.20732

**Published:** 2017-09-08

**Authors:** Yiquan Xiong, Qiuyang Zhao, Zhi Li, Shuntao Wang, Hui Guo, Zeming Liu, Tao Huang

**Affiliations:** ^1^ Department of Breast and Thyroid Surgery, Union Hospital, Tongji Medical College, Huazhong University of Science and Technology, Wuhan 430022, People's Republic of China

**Keywords:** oxyphilic thyroid carcinoma, prognosis, SEER, PSM

## Abstract

There is controversy regarding the prognosis of patients with oxyphilic thyroid cancer (OXTC). The present study compared the prognoses of OXTC, papillary thyroid cancer (PTC), and follicular thyroid cancer (FTC), in order to provide a new perspective regarding the treatment guidelines for these diseases. We evaluated data from patients with thyroid cancer who were included in the Surveillance, Epidemiology, and End Results database between 2004 and 2013. Patient mortality was evaluated using Cox proportional hazards regression analyses and Kaplan-Meier analyses with log-rank tests. The multivariate Cox regression analysis revealed that the cancer-specific survival rate for OXTC was similar to that for PTC, but higher than that for FTC. However, after propensity score matching for relevant factors, the cancer-specific survival rate for OXTC was higher than that for PTC and FTC. This unexpected result provides new implications for the treatment of patients with OXTC.

## INTRODUCTION

The incidence of thyroid cancer has risen rapidly during recent decades [1–3]. Papillary thyroid cancer (PTC) and follicular thyroid cancer (FTC) (i.e., differentiated thyroid cancer) account for >90% of all thyroid malignancies and are the most common types of thyroid carcinoma [1, 4, 5]. However, there are other rare histological variants of thyroid cancer, such as tall cell, solid, or oxyphilic thyroid cancer (OXTC) [6–8]. OXTC is also known as Hurthle cell carcinoma, and only accounts for 2–5% of all thyroid cancers [9–11]. The current World Health Organization classification system has defined OXTC as a variant of FTC, although Ganly et al. have reported that OXTC may be distinct from PTC and FTC, based on the unique genetic features of OXTC [12]. Moreover, given the rarity of OXTC, few studies have evaluated its clinical characteristics and optimal treatment [11]. The present study compared the prognoses (cancer-specific and all-cause mortality) of OXTC, PTC, and FTC, using data from the Surveillance, Epidemiology, and End Results (SEER) database (2004–2013) and propensity score matching (PSM).

## RESULTS

### Demographic and clinical features

This study evaluated data from 101,443 patients with thyroid cancer, including 2,615 patients with OXTC, 92,963 patients with PTC, and 5,865 patients with FTC. The patients’ mean age and follow-up duration according to histological subtype are shown in Table 1. Patients with OXTC were significantly older, compared to patients with PTC or FTC.

**Table 1:** Characteristics for Patients with different histological types

Covariate	level	Histological types				
		OXTC (n=2615)	PTC (n=92963)	P value	FTC (n=5865)	P value
Age (year)		57.69±15.68	49.36±15.29	<0.001	51.33±17.20	<0.001
Sex	Female	1813(69.3%)	71785(77.2%)	<0.001	4137(70.5%)	0.262
	Male	802(30.7%)	21178(22.8%)		1728(29.5%)	
Race	White	2201(85.1%)	76038(82.9%)	<0.001	4529(78.3%)	<0.001
	Black	222(8.6%)	5704(6.2%)		695(12.0%)	
	Other	164(6.3%)	9987(10.9%)		558(9.7%)	
T stage	T1	615(25.0%)	55606(62.2%)	<0.001	1283(23.8%)	<0.001
	T2	868(35.4%)	13811(15.5%)		2174(40.2%)	
	T3	838(34.1%)	16407(18.4%)		1747(32.4%)	
	T4	135(5.5%)	3463(3.9%)		194(3.6%)	
N-stage	N0	2355(95.2%)	69465(79.0%)	<0.001	5370(97.0%)	<0.001
	N1	120(4.8%)	18460(21.0%)		168(3.0%)	
M-stage	M0	2453(96.6%)	89658(98.8%)	<0.001	5377(94.2%)	<0.001
	M1	87(3.4%)	1132(1.2%)		329(5.8%)	
Multifocality	No	2033(82.3%)	52324(58.3%)	<0.001	4735(85.6%)	<0.001
	Yes	438(17.7%)	37350(41.7%)		797(14.4%)	
Extension	No	2222(87.1%)	75994(83.7%)	<0.001	5114(90.5%)	<0.001
	Yes	329(12.9%)	14847(16.3%)		539(9.5%)	
Radiation	None or refused	1094(42.9%)	46761(51.5%)	<0.001	2573(45.0%)	0.109
	Radiation Beam or Rdioactive implants	94(3.7%)	1705(1.9%)		177(3.1%)	
	Radioisotopes or Radiation beam plus isotopes or implants	1364(53.4%)	42296(46.6%)		2970(51.9%)	
Surgery	Lobectomy	469(18.9%)	12688(14.2%)	<0.001	1300(23.6%)	<0.001
	Subtotal or near-total thyroidectomy	147(5.9%)	3337(3.7%)		303(5.5%)	
	Total thyroidectomy	1871(75.2%)	73162(82.1%)		3911(70.9%)	
Survival months (month)		53.90±34.45	49.09±33.76	<0.001	53.12±34.33	0.331

OXTC: Oxyphilic thyroid adenocarcinoma; PTC: papillary thyroid cancer; FTC: follicular thyroid carcinoma.

### Cancer-specific and all-cause mortality rates

During the follow-up period, cancer-specific death was detected for 101 patients in the OXTC group, 996 patients in the PTC group, and 190 patients in the FTC group. The cancer-specific mortality rates per 1,000 person-years were 8.343 (95% confidence interval [CI]: 6.844–10.169) for OXTC, 2.403 (95% CI: 2.252–2.564) for PTC, and 6.509 (95% CI: 5.598–7.569) for FTC (Table 2). In addition, during the follow-up period, all-cause death was detected for 319 patients in the OXTC group, 4,388 patients in the PTC group, and 538 patients in the FTC group. The all-cause mortality rates per 1,000 person-years were 26.646 (95% CI: 23.852–29.768) for OXTC, 11.068 (95% CI: 10.739–11.408) for PTC, and 19.337 (95% CI: 17.717–21.104) for FTC (Table 2).

**Table 2:** Hazard Ratios of different histological types for the cancer specific deaths and all cause deaths of thyroid cancer

Histological types	Cancer-Specific Deaths,	%	Cancer-Specific Deaths per	95% CI	All Cause Deaths,	%	All Cause Deaths per	95% CI
	No.		1,000 Person-Years		No.		1,000 Person-Years	
OXTC	101	3.86	8.343	6.844-10.169	319	12.19	26.646	23.852-29.768
PTC	966	1.04	2.403	2.252-2.564	4388	4.72	11.068	10.739-11.408
FTC	190	3.24	6.509	5.598-7.569	538	9.17	19.337	17.717-21.104

OXTC: Oxyphilic thyroid adenocarcinoma; PTC: papillary thyroid cancer; FTC: follicular thyroid carcinoma.

### Risk factors for cancer-specific and all-cause mortality

The univariate Cox regression analyses revealed that cancer-specific mortality was associated with age, sex, race, histological type, TNM stage, extension, radiation treatment, and surgical approach. The multivariate Cox regression model revealed that PTC was an independent risk factor for cancer-specific mortality, compared to OXTC (Table 3). The univariate Cox regression analyses revealed that all-cause mortality was associated with age, sex, race, histological type, TNM stage, multifocality, extension, radiation treatment, and surgical approach. The multivariate Cox regression model revealed that PTC was also an independent risk factor for all-cause mortality, compared to OXTC (Table 3).

**Table 3:** Risk factors for survival: outcome of thyroid cancer specific mortality and all-cause mortality

Covariate	level	Thyroid Cancer specific mortality				All cause mortality			
		Univariate Cox regression		Multivariate Cox regression		Univariate Cox regression		Multivariate Cox regression	
		Hazard Ratio (95% CI)	p-value	Hazard Ratio (95% CI)	p-value	Hazard Ratio (95% CI)	p-value	Hazard Ratio (95% CI)	p-value
Age		1.097(1.093-1.102)	<0.001	1.067(1.061-1.072)	<0.001	1.087(1.085-1.089)	<0.001	1.077(1.074-1.080)	<0.001
Sex	Female	ref		ref		ref		ref	
	Male	2.673(2.392-2.988)	<0.001	1.332(1.143-1.553)	<0.001	2.439(2.308-2.576)	<0.001	1.634(1.526-1.750)	<0.001
Race	White	ref		ref		ref		ref	
	Black	1.075(0.860-1.343)	0.525	1.147(0.826-1.591)	0.413	1.286(1.165-1.420)	<0.001	1.426(1.261-1.612)	<0.001
	Other	1.428(1.217-1.675)	<0.001	0.921(0.735-1.154)	0.473	0.941(0.858-1.032)	0.199	0.802(0.710-0.906)	<0.001
Histological types	OXTC	ref		ref		ref		ref	
	PTC	0.288(0.234-0.353)	<0.001	0.787(0.583-1.063)	0.118	0.423(0.377-0.474)	<0.001	0.811(0.701-0.938)	0.005
	FTC	0.850(0.668-1.082)	0.186	1.489(1.056-2.099)	0.023	0.763(0.664-0.876)	<0.001	1.020(0.858-1.212)	0.824
T-stage T-stage	T1	ref		ref		ref		ref	
	T2	2.807(2.123-3.712)	<0.001	2.020(1.445-2.823)	<0.001	1.128(1.033-1.232)	0.007	1.104(0.997-1.224)	0.058
	T3	8.251(6.609-10.300)	<0.001	3.789(2.705-5.307)	<0.001	1.683(1.561-1.814)	<0.001	1.220(1.072-1.388)	0.003
	T4	86.971(70.791-106.849)	<0.001	13.626(9.256-20.058)	<0.001	7.737(7.160-8.361)	<0.001	2.723(2.285-3.244)	<0.001
N stage	N0	ref		ref		ref		ref	
	N1	4.533(4.011-5.123)	<0.001	2.128(1.782-2.541)	<0.001	1.635(1.532-1.746)	<0.001	1.542(1.408-1.689)	<0.001
M-stage	M0	ref		ref		ref		ref	
	M1	49.059(43.578-55.229)	<0.001	6.345(5.282-7.621)	<0.001	13.482(12.423-14.631)	<0.001	3.730(3.272-4.251)	<0.001
Multifocality	No	ref		ref		ref		ref	
	Yes	0.883(0.777-1.002)	0.055	0.782(0.670-0.914)	0.002	0.862(0.812-0.915)	<0.001	0.962(0.897-1.033)	0.290
Extension	No	ref		ref		ref		ref	
	Yes	12.707(11.172-14.452)	<0.001	1.517(1.124-2.049)	0.007	2.634(2.480-2.798)	<0.001	1.093(0.946-1.264)	0.228
Radiation	None or refused	ref		ref		ref		ref	
	Radiation Beam or Rdioactive implants	14.719(12.728-17.022)	<0.001	2.754(2.185-3.472)	<0.001	3.594(3.247-3.979)	<0.001	1.409(1.212-1.638)	<0.001
	Radioisotopes or Radiation beam+ isotopes/implants	0.895(0.787-1.018)	0.91	0.814(0.679-0.976)	0.026	0.580(0.546-0.615)	<0.001	0.693(0.643-0.748)	<0.001
Surgery	Lobectomy	ref		ref		ref		ref	
	Subtotal or near-total thyroidectomy	2.009(1.471-2.744)	<0.001	1.339(0.903-1.985)	0.147	1.047(0.908-1.209)	0.527	1.047(0.890-1.230)	0.581
	Total thyroidectomy	1.332(1.087-1.633)	0.006	1.127(0.868-1.462)	0.370	0.803(0.743-0.869)	<0.001	0.960(0.875-1.052)	0.381

OXTC: Oxyphilic thyroid carcinoma; PTC: papillary thyroid cancer; FTC: follicular thyroid carcinoma.

### Adjusting for patient characteristics using PSM

**Figure 1:**
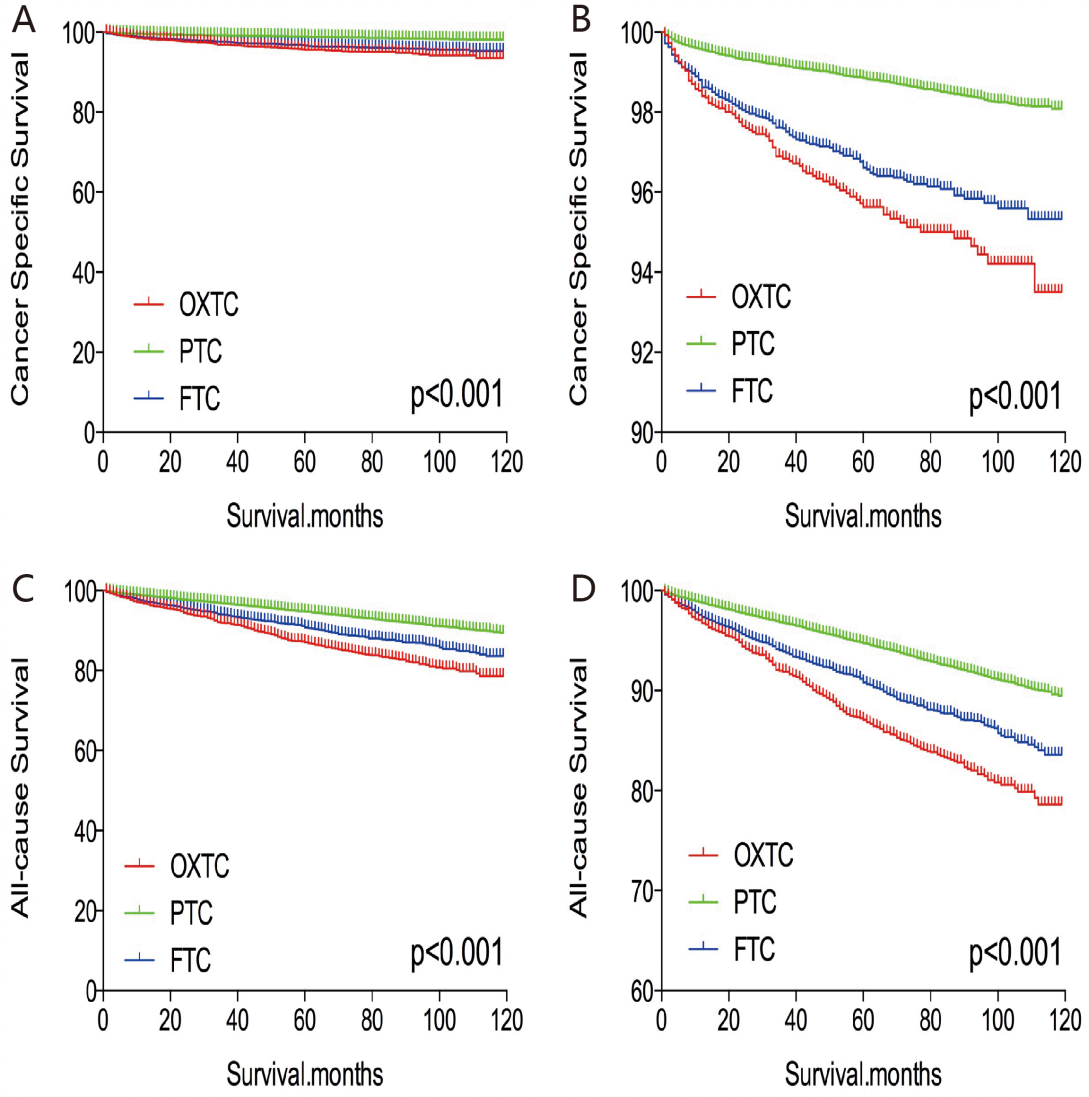
Kaplan Meier curves among patients stratified by subtype for cancer-specific mortality **(A, B)** and all cause mortality **(C, D)**.

**Figure 2:**
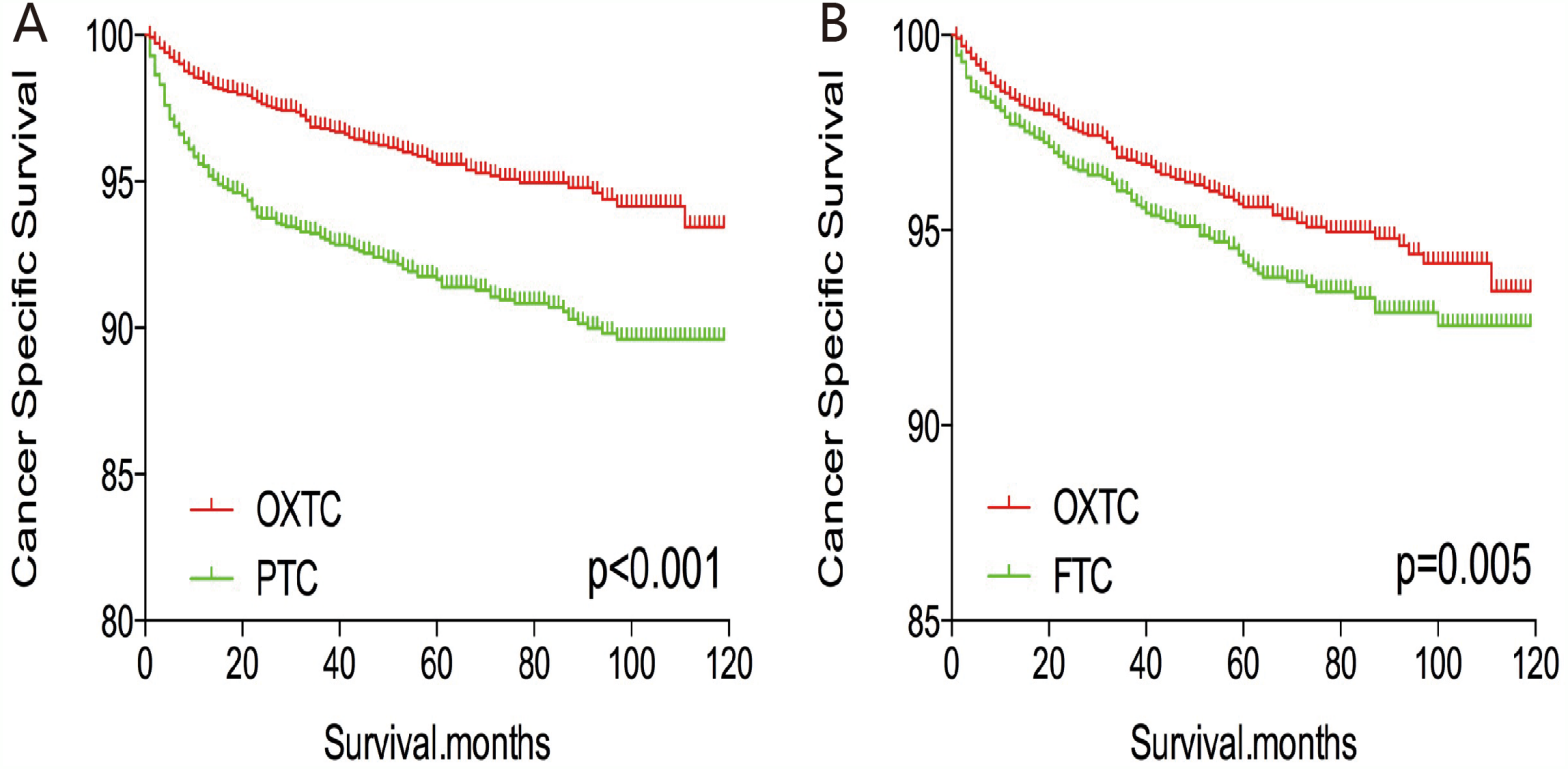
Kaplan Meier curves of cancer-specific mortality for matched subtype pairs Age, sex and race matching between OXTC and PTC **(A)**, OXTC and FTC **(B)**.

**Figure 3:**
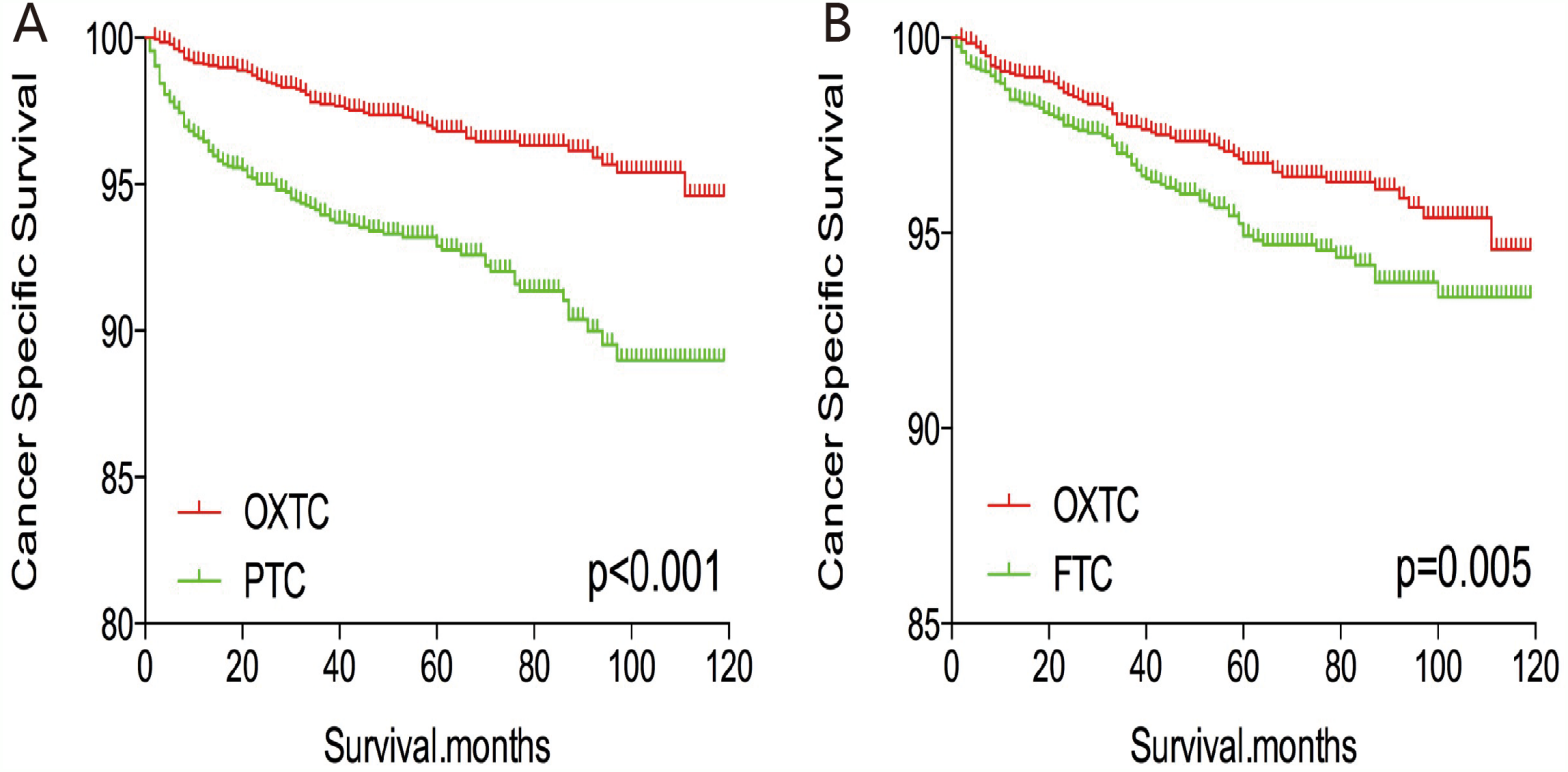
Kaplan Meier curves of cancer-specific mortality for matched Subtype pairs Age, sex, race, T/N/M stage, multifocality, extension matched between OXTC and PTC **(A)**, OXTC and FTC **(B)**.

**Figure 4:**
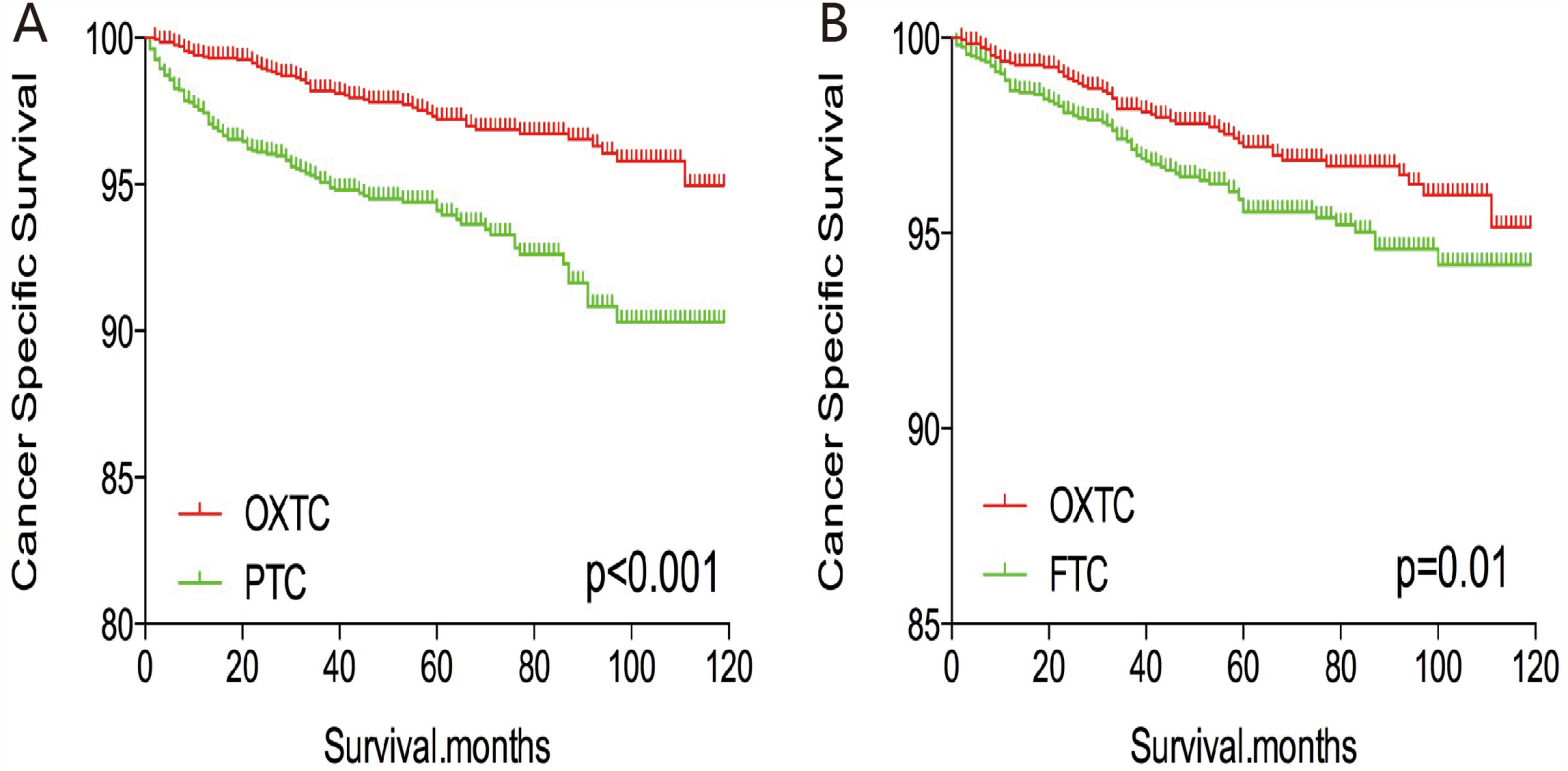
Kaplan Meier curves of cancer-specific mortality for matched Subtype pairs Age, sex, race, T/N/M stage, multifocality, extension, surgery and radiation treatment matched between OXTC and PTC **(A)**, OXTC and FTC **(B)**.

The cancer-specific and all-cause mortality rates were significantly different when we compared OXTC to PTC and FTC (both p < 0.001, Figure 1A–1D). Thus, to minimize selection bias, PSM was performed for age, sex, race, TNM stage, multifocality, extension, and radiation treatment. After PSM for age, sex, and race, OXTC was associated with a lower cancer-specific mortality rate, compared to PTC (p < 0.001) and FTC (p = 0.005) (Figure 2A, 2B). After PSM for age, sex, race, TNM stage, multifocality, and extension, OXTC was still associated with a significantly lower cancer-specific mortality rate, compared to PTC (p < 0.001) and FTC (p = 0.005) (Figure 3A, 3B). After PSM for all relevant factors and radiation and surgery treatment, the cancer-specific mortality rate for OXTC remained significantly lower than that for PTC (p < 0.001) and FTC (p = 0.01) (Figure 4A, 4B).

**Figure 5:**
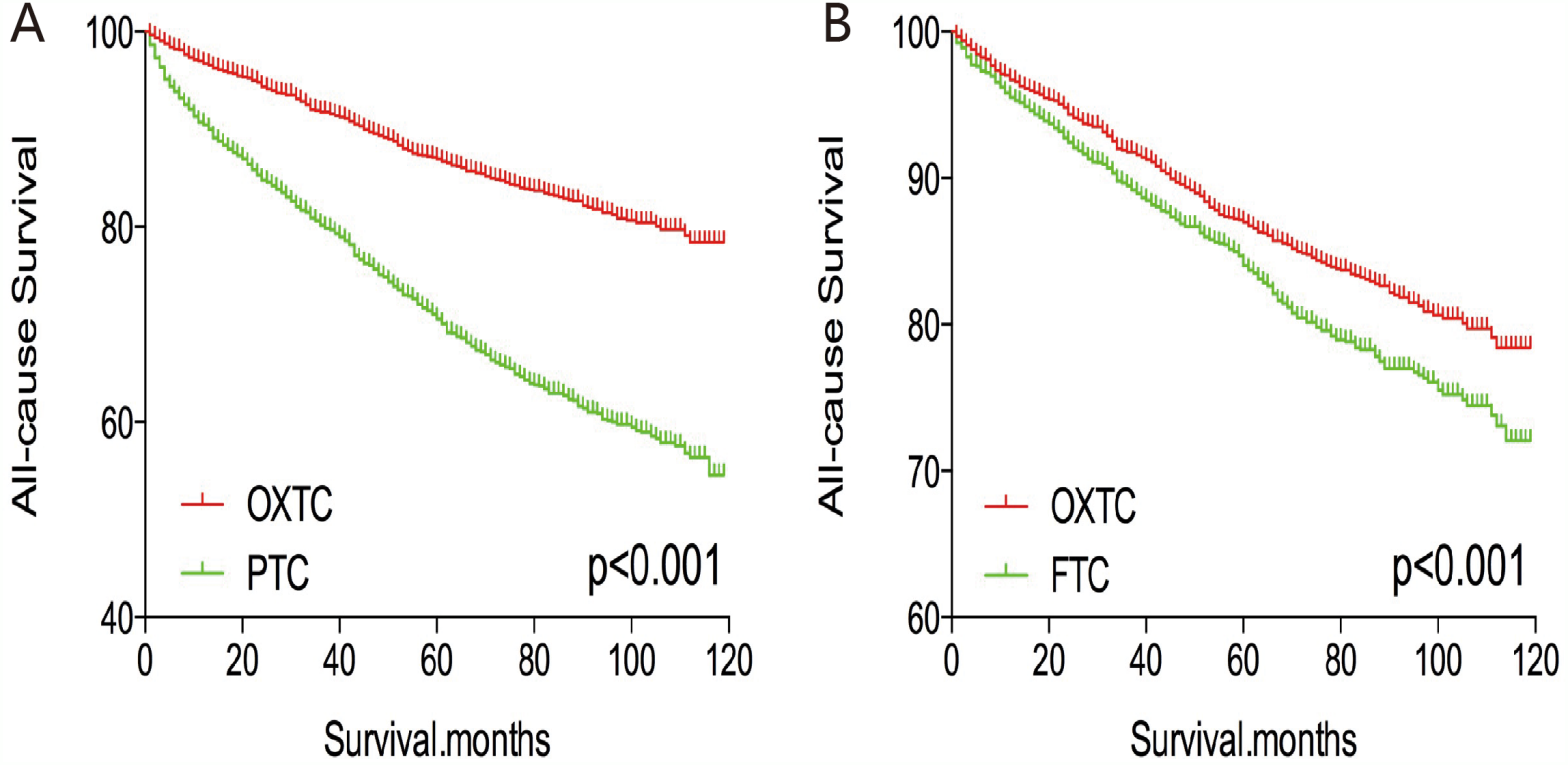
Kaplan Meier curves of all cause mortality for matched Subtype pairs Age, sex and race matching between OXTC and PTC **(A)**, OXTC and FTC **(B)**.

**Figure 6:**
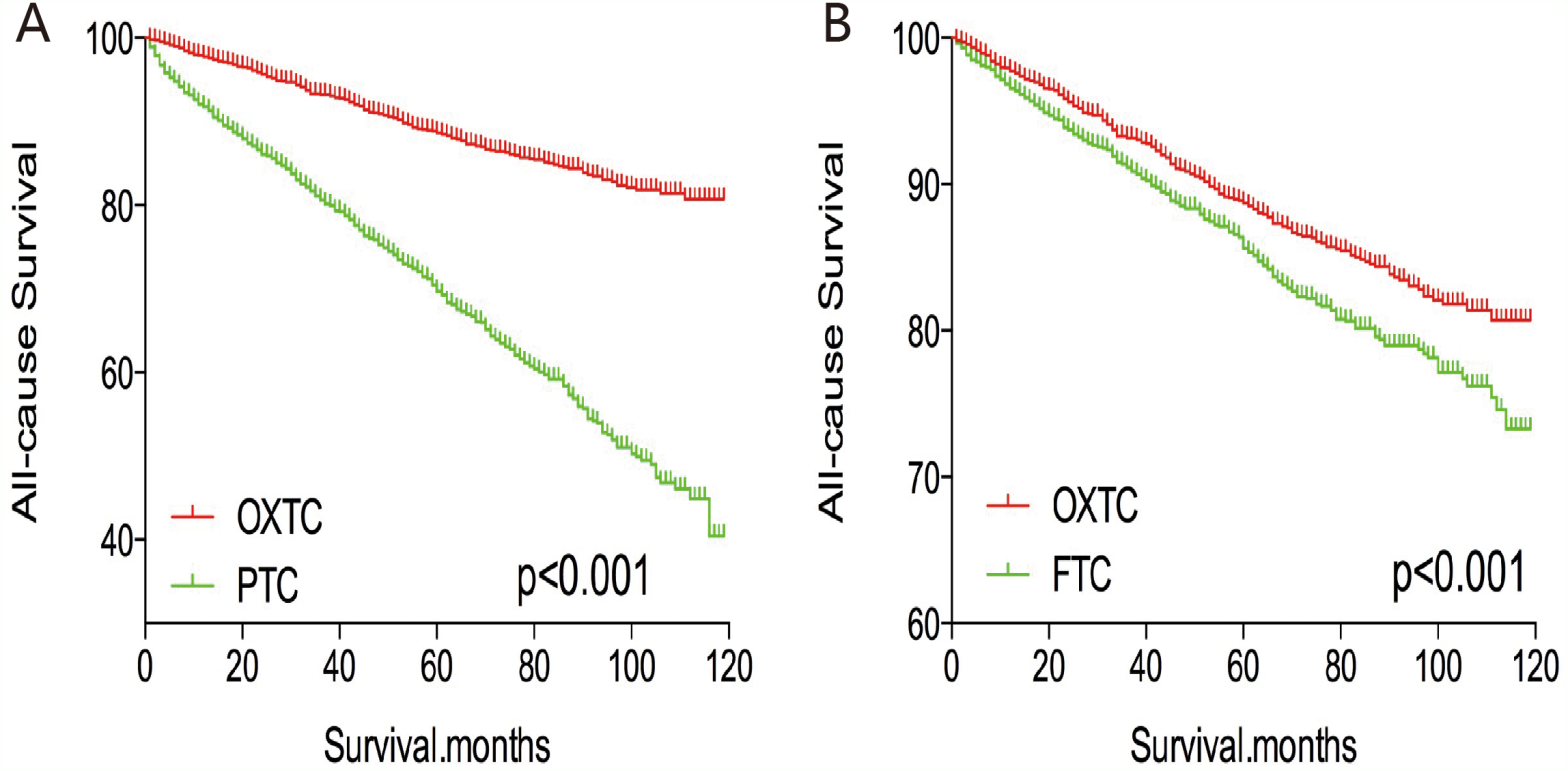
Kaplan Meier curves of all cause mortality for matched Subtype pairs Age, sex, race, T/N/M stage, multifocality, extension matching between OXTC and PTC **(A)**, OXTC and FTC **(B)**.

**Figure 7:**
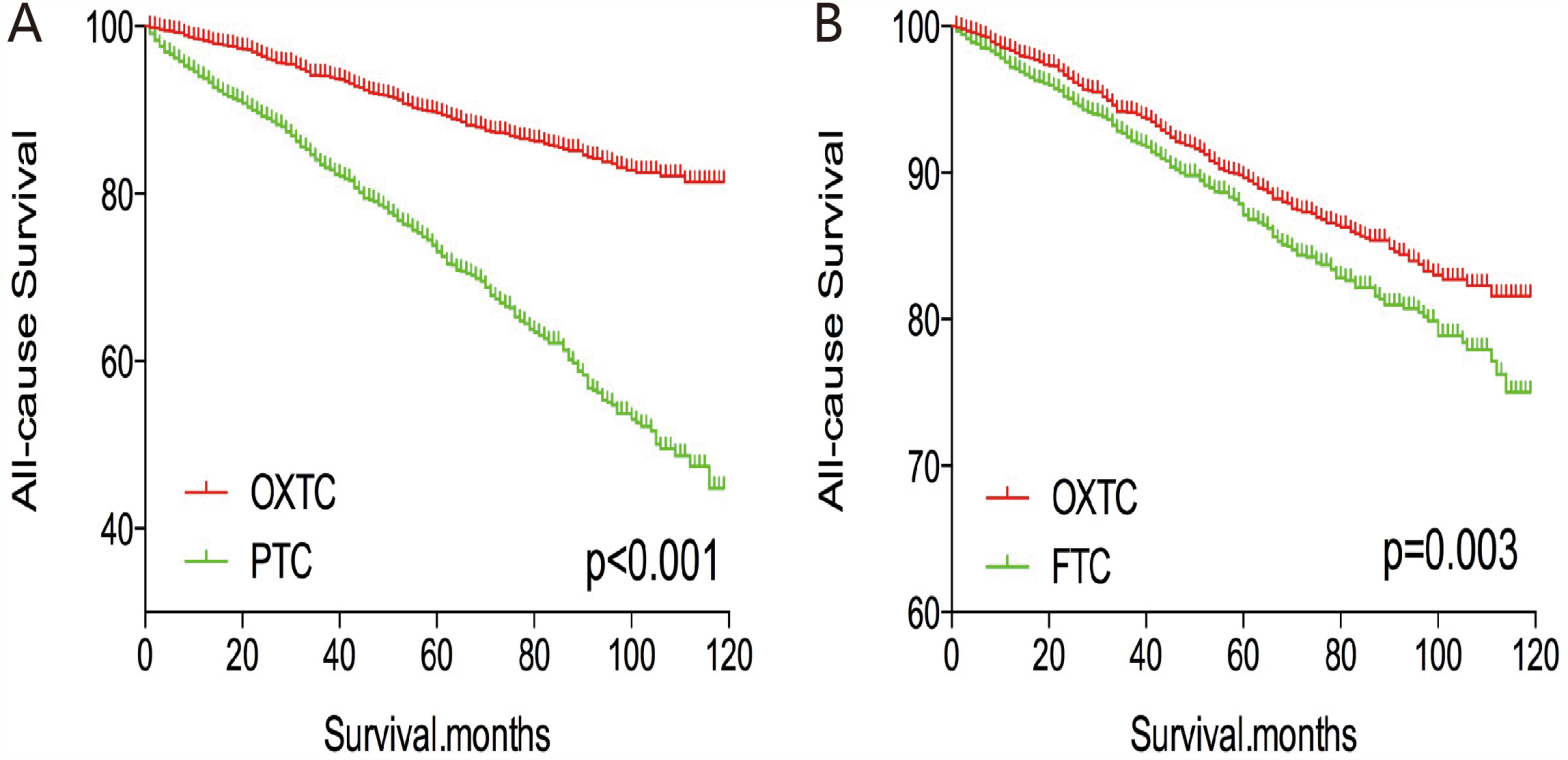
Kaplan Meier curves of all cause mortality for matched Subtype pairs Age, sex, race, T/N/M stage, multifocality, extension, surgery and radiation treatment matching between OXTC and PTC **(A)**, OXTC and FTC **(B)**.

After PSM for age, sex, and race, OXTC was associated with a lower all-cause mortality rate, compared to PTC and FTC (both p < 0.001, Figure 5A, 5B). Similar results were obtained after PSM for age, sex, race, TNM stage, multifocality, and extension (Figure 6A, 6B). After PSM for all relevant factors and radiation and surgery treatment, OXTC was associated with a lower all-cause mortality rate, compared to PTC (p < 0.001) and FTC (p = 0.003) (Figure 7A, 7B).

## DISCUSSION

Oxyphilic thyroid tumors (Hurthle cell tumors) include Hurthle cell adenoma, Hurthle cell thyroid carcinoma, and Hurthle cell papillary thyroid carcinoma. The oxyphilic cells exhibit various features, such as a finely granular eosinophilic cytoplasm and an increased number of mitochondria in the thyroid ultrastructure [13]. However, given the rarity of thyroid malignancies, little is known regarding the long-term survival of patients with OXTC, and conflicting results have been reported in the limited number of single-center studies, which had relatively small samples sizes [14–17].

In contrast, the SEER database identifies and tracks patients from diverse geographic regions, and is considered the gold standard database for tumor surveillance and survival analysis in the US, as it contains data from approximately 10% of the American patient population [18]. Therefore, the present study evaluated the prognosis of OXTC using population-based data from the SEER database.

Goffredo et al. evaluated SEER data (1998–2009) and reported that OXTC was more aggressive and had a poorer prognosis, compared to the other types of differentiated thyroid cancer [19]. However, that study did not compare the prognoses of OXTC and other thyroid cancers using PSM, and may have been limited by its inability to adjust for confounding factors.

In the present study, patients with OXTC were older (57.69 years old), compared to patients with PTC and FTC. Many studies have indicated that age plays a unique prognostic role in thyroid cancer [18, 20, 21], and the present study confirmed that age was an independent risk factor for both cancer-specific and all-cause mortality. Furthermore, PSM for age, race, and sex confirmed that OXTC was associated with a better prognosis, compared to PTC and FTC. Therefore, an older age at the diagnosis of OXTC may be associated with a relatively poor prognosis.

Previous reports have provided conflicting information regarding whether OXTC has more aggressive clinical features, compared to PTC or FTC [22–25]. However, it is clear that aggressive clinical characteristics (e.g., nodal metastasis, distant metastasis, and larger tumor size) are associated with more advanced disease and shorter cancer-specific survival. In the present study, PSM for demographic and clinical risk factors revealed that OXTC was associated with lower cancer-specific and all-cause mortality rates, compared to PTC and FTC.

The main purpose of radioiodine (RAI) treatment after total thyroidectomy is the ablation of residual thyroid tissue, which facilitates the early detection of cancer recurrence [26]. However, the current guidelines debate the use of RAI for OXTC [11]. In the present study, patients with OXTC were more likely to receive RAI treatment, compared to patients with PTC and FTC, although there remains insufficient evidence regarding the effectiveness of RAI treatment in this setting.

Ganly et al. have indicated that OXTC may be a unique type of thyroid malignancy, compared to PTC and FTC, as it has unique mutational, transcriptional, cytogenetic, and gene expression changes [12]. For example, OXTC was not associated with alterations in the *BRAF*, *PIK3CA*, or *PPAR* genes, and only 11.1% of OXTC cases involved *RAS* mutations. Although the Wnt/β-catenin pathway is considered highly activated in OXTC, the SEER database does not contain mutation information and we could not include genetic status in our analyses.

There are several limitations in the present study. First, the SEER database lacks information regarding recurrence and surgery-related comorbidities, and we could not account for these factors in our analyses. Second, we did not evaluate or consider the patients’ family history, vascular invasion status, accurate extension information or other histological characteristics. Third, the SEER database does not include information regarding whether the patients underwent repeat surgery, and this lack of information may have biased our findings.

In conclusion, our results indicate that patients who were diagnosed with OXTC had an unexpectedly good prognosis, compared to patients with PTC or FTC. This information may be useful for selecting appropriate treatments for these patients.

## MATERIALS AND METHODS

### Ethical considerations and data collection

This study's retrospective protocol was approved by our institution's ethical review board and complied with the ethical standards of the Declaration of Helsinki, as well as the relevant national and international guidelines. The SEER project is an American population-based cancer registry that was launched in 1973 and is supported by the Centers for Disease Control and Prevention and the National Cancer Institute. The database contains information regarding cancer incidence, prevalence, and mortality, as well as population-based variables and primary tumor characteristics (i.e., histological subtype) from multiple geographic regions.

The present study included and evaluated SEER data (2004–2013) from patients with thyroid cancer according to their subtype (OXTC, PTC, and FTC) using code C73.9 from the International Classification of Diseases for Oncology (i.e., thyroid, papillary, and/or follicular histology). The eligible diagnostic codes were: “papillary carcinoma”, “papillary adenocarcinoma”, “oxyphilic adenocarcinoma”, “follicular adenocarcinoma”, “papillary carcinoma, follicular variant”, and “papillary & follicular adenocarcinoma”. Cases without American Joint Committee on Cancer staging information (version 6) were excluded to ensure accurate analyses. Cases without information of follow up time were also excluded. The three histological subtypes were compared according to age, sex, race, TNM stage, multifocality, extension, and radiation treatment (i.e., none or refused, external beam radiation therapy, or RAI).

### Statistical analyses

The quantitative variables were expressed as mean ± standard deviation (SD), while the categorical ones were presented as percentages. Kaplan-Meier survival curves with the log-rank test were used for determination of all-cause survival and cancer-specific survival. Cox proportional hazard regression analyses were using to estimate hazard ratios and 95% CIs, in order to quantify the effects of the different histological subtypes on cancer-specific and all-cause mortality. PSM was also used to further adjust for potential baseline confounding factors. All p-values were 2-sided, and p-values < .05 were considered significant. Analyses were performed using SPSS version 23.0, Stata/SE version 12 (Stata Corp.), and GraphPad Prism version 6 (GraphPad Software Inc.).

## Abbreviations

CPTC: classic papillary thyroid cancer
CI: confidence interval
FTC: follicular thyroid cancer
OXTC: oxyphilic thyroid carcinoma
PSM: propensity score matching
PTC: papillary thyroid cancer
SEER: Surveillance, Epidemiology, and End Results
