# Correction: Propensity score matching analysis of the prognosis for the rare oxyphilic subtype of thyroid cancer (Hurthle cell carcinoma)

**DOI:** 10.18632/oncotarget.25000

**Published:** 2018-03-23

**Authors:** Yiquan Xiong, Qiuyang Zhao, Zhi Li, Shuntao Wang, Hui Guo, Zeming Liu, Tao Huang

**Affiliations:** ^1^ Department of Breast and Thyroid Surgery, Union Hospital, Tongji Medical College, Huazhong University of Science and Technology, Wuhan 430022, China

**This article has been corrected:** The proper name of the institution is as follows:

^1^ Department of Breast and Thyroid Surgery, Union Hospital, Tongji Medical College, Huazhong University of Science and Technology, Wuhan 430022, China

Original article: Oncotarget. 2017; 8:101362-101371. https://doi.org/10.18632/oncotarget.20732

